# Memory-Enhancing and Anxiolytic Effects of the Rose of Jericho on Sleep Deprivation-Related Cognitive and Behavioral Changes

**DOI:** 10.7759/cureus.78327

**Published:** 2025-02-01

**Authors:** Salah A Mustafa, Joud Alsaeed, Eman M Alyaseen, Roba A Alhazmi, Renad A Alhazmi, Mazen S Alzahrani, Nouran M Almehmadi, Farah A Al Ali, Salman S Salman, Amar M Marwani, Mariwan Husni, Yahya M Naguib

**Affiliations:** 1 College of Medicine and Health Sciences, Arabian Gulf University, Manama, BHR; 2 Family Medicine Department, Anak General Hospital, Ministry of Health of Saudi Arabia, Dammam, SAU; 3 Animal House Unit, College of Medicine and Health Sciences, Arabian Gulf University, Manama, BHR; 4 Psychiatry Department, Northern Ontario School of Medicine University, Ontario, CAN; 5 Psychiatry Department, College of Medicine and Health Sciences, Arabian Gulf University, Manama, BHR; 6 Physiology Department, College of Medicine and Health Sciences, Arabian Gulf University, Manama, BHR; 7 Clinical Physiology Department, Faculty of Medicine, Menoufia University, Shebin El Kom, EGY

**Keywords:** behavior, cognition, memory, rose of jericho, sleep deprivation, stress

## Abstract

Background: Sleep is a crucial physiological phenomenon that enables the body to engage in restoration and rejuvenation. Remarkably, even limited periods of sleep deprivation (SD) can adversely affect cognitive functions such as memory retention, emotional regulation, data processing, and concentration. The Rose of Jericho (RoJ) has been considered more than a plant and has demonstrated potential therapeutic actions in childbirth, respiratory diseases, gastrointestinal disorders, and cancer. The effect of the RoJ on memory, cognition, and behavior has not yet been well-studied.

Objectives: The present study aimed to investigate the possible therapeutic effects of the RoJ on memory, cognition, behavior, and motor coordination in a rat model of SD.

Materials and methods: Thirty male Wistar albino rats weighing 120-150 g were used in the present study. The rats were acclimatized and trained and then randomly divided into three groups: control (C), sleep-deprived (SD), and SD treated with RoJ (SD+RoJ). Spatial memory and learning were assessed using the Morris Water Maze (MWM) test, while anxiety-related behaviors were evaluated through the Elevated Plus Maze (EPM) test. The rotarod test was used to assess motor coordination.

Results: The study revealed significant behavioral and cognitive performance improvements with the SD+RoJ group across all the tests. In the MWM test, the SD group exhibited a marked increase in test duration (29.5 ± 3.57 sec) and a reduction in average speed (1.9 ± 0.3 cm/s) when compared to the C group (13.41 ± 1.57 sec and 5.9 ± 0.34 cm/s, respectively). Interestingly, the SD+RoJ group significantly reduced test duration (19.75 ± 3.36 sec) and improved rats’ speed (6.06 ± 0.27 cm/s) compared to the SD group. The EPM test demonstrated that the SD group spent significantly less time in the open arms (16.2 ± 9.44 sec) than the C group (59.8 ± 3.29 sec). Interestingly, the SD+RoJ group significantly improved the time spent in the open arms (45.8 ± 11.64 sec). Moreover, the SD+RoJ group showed notable improvement in open-arm entries (7 ± 2.39) compared to the SD group (1.6 ± 0.81). In the Rotarod test, the SD group demonstrated a significant decline in latency to fall (44.2 ± 9.5 sec) compared to the C group (228.67 ± 35.44 sec). The SD+RoJ group exhibited a significantly longer falling latency (165 ± 28.77 sec) than the SD group.

Conclusion: Treatment with the RoJ alleviated SD-dependent cognitive impairment, anxiety, and decline in motor coordination. Supplementation with the RoJ may offer potential therapeutic benefits, including boosting memory, improving cognition, reducing anxiety and depression, and enhancing motor coordination.

## Introduction

Sleep is a complex interplay of multiple physiological processes predominantly governed by neurobiological mechanisms [1]. These mechanisms are evident at genetic, biological, and cellular organizational levels. Several brain areas regulate sleep, including the basal forebrain, thalamus, and hypothalamus. Serotonin, norepinephrine, histamine, orexin, acetylcholine, dopamine, glutamate, and gamma-aminobutyric acid control sleep neurobiological mechanisms [2]. Sleep is governed by a finely tuned interplay of quantitative and qualitative parameters. Disruption of these regulatory mechanisms can precipitate sleep deprivation (SD). This condition has become increasingly prevalent across all age groups and diverse populations in the context of modern, high-pressure lifestyles [3]. The etiology of SD involves interrelated medical, psychosocial, and occupational stressors, which contribute to its global impact on public health [4]. SD has profound effects on health, contributing to various physiological and psychological disorders, including an increased risk of cardiovascular diseases, obesity, diabetes, and weakened immune function [5]. Among medical students, SD is a critical concern, often stemming from academic pressures, social commitments, and increased screen time, leading to decreased cognitive functions, emotional instability, and heightened stress levels [6]. Furthermore, SD significantly impacts healthcare professionals, with approximately 50% of physicians reporting inadequate sleep, which contributes to a 30% increase in medical errors and a 20% decrease in patient satisfaction, underscoring the need to address SD to improve both staff well-being and patient care quality [7,8].

Herbal medicine has long been a cornerstone of traditional healthcare across diverse cultures and regions, with its use passed down through generations [9]. Among these, *Anastatica hierochuntica* (Rose of Jericho (RoJ), resurrection plant, Genggam Fatimah, Kaf Mariam) stands out due to its rich composition of bioactive compounds, including chlorogenic acid, quercetin, luteolin, and kaempferol. These compounds possess antioxidant, anti-inflammatory, and neuroprotective properties, offering significant therapeutic potential for treating conditions such as oxidative stress, hyperuricemia, gout, and renal impairment due to their anti-inflammatory and antioxidant properties [10-12]. Furthermore, in various regions, RoJ has been traditionally used for wound healing, immune system support, and treating reproductive system-related disorders [12,13]. Its medicinal value continues to gain attention in modern medical research.

There is growing interest in identifying natural interventions that can mitigate the detrimental effects of sleep deficiency on cognitive performance and oxidative balance. Little is known about the neuroprotective effects of RoJ. The present study investigated the potential therapeutic effects of RoJ in counteracting SD-dependent memory impairment and behavioral changes in experimental animals.

## Materials and methods

Ethical considerations

This research was conducted in accordance with the regulations and guidelines set forth by the Research and Ethics Committee of Arabian Gulf University (approval number: E23-PI-1-23), as well as the Guiding Principles for the Use and Care of Animals [14]. All personnel involved in the laboratory procedures were trained and certified in handling laboratory animals. At the end of the experiment, all rats were sacrificed by CO2 inhalation.

Experimental settings and animals

This study was performed between September 01, 2023, and June 30, 2024, at the Animal House Unit of the Faculty of Medicine and Health Sciences, Arabian Gulf University, Kingdom of Bahrain. Thirty male Wistar albino rats weighing 150-200 g were allowed to acclimatize for 10 days and were fed standard laboratory chow ad libitum. Rats were also given free access to water in an air-conditioned room with a 12-hour light/dark cycle.

Study groups

Rats were randomly and equally assigned to three experimental groups as follows: control (C) group (rats were housed in their cages with normal sleep cycles and unrestricted access to food and water), sleep deprivation (SD) group (rats underwent 72 hours of paradoxical SD and received a daily morning single dose of 1 ml distilled water via gastric lavage), and SD RoJ-treated (SD+RoJ) group (rats were subjected to 72 hours of paradoxical SD with concomitant administration of RoJ in a daily morning single dose of 250 mg/kg (1 ml) via gastric lavage (5% extract, Dreams Oil presses, Jeddah, SA) [10].

Induction of sleep deprivation

SD was induced as described previously [12,15]. The rats were housed in a specially designed glass tank (120 x 40 x 40 cm) with 10 platforms. The platforms were designed to enable the rats to remain alert and stand comfortably while preventing them from falling asleep. If the rats were to fall asleep, they would fall in the water and wake up. Consequently, the rats can only move by jumping between the platforms. Rats were placed in the empty glass tank before filling it with water for one hour/day for three days prior to the start of the experiment to allow proper acclimatization. Ultimately, the tank was filled with water 3 cm below the surface of the platforms.

Blinding of outcome assessors

Blinding of assessors was maintained to minimize bias and maximize the validity of the study results. All possible measures to minimize stress before/during induction of SD or outcome assessments were taken.

Assessment of spatial memory and learning

Spatial memory and learning were assessed using the Morris Water Maze (MWM) test as previously described [16]. Briefly, rats were placed in an opaque pool and were required to swim to a hidden escape platform. Following a five-day training program (after the acclimatization period and before the SD period), rats became more familiar with the test, and both their traveled distance and speed in finding the hidden platform improved. The following metrics were recorded: total test duration (sec), average speed (cm/sec), and total traveled distance (cm).

Assessment of anxiety-related behavior

Anxiety-related behavior was assessed using the Elevated Plus Maze (EPM) test as described previously [17]. In brief, the EPM configuration provides two open and two closed arms arranged in a cross shape. The ratio of the time spent in the open arms (sec) and the time spent in the closed arms (sec), as well as the number of entries into the open arms, were considered as a reflection of the rats’ anxiety-related behavior. Rats were trained for five consecutive days following the acclimatization period and prior to performing the recorded experiment.

Assessment of motor function and coordination

Motor function and coordination were assessed with the rotarod test [18]. The rotarod evaluates motor function and coordination by examining neural centers like the cerebellum and sensorimotor cortex. Rats were placed on a rotating rod that increased speed. The latency (time until rats fall off) was measured to assess the impact of stress on motor coordination.

Statistical analysis

Origin Pro 2023 software (OriginLab Corporation, MA, USA) was used to analyze the data generated from the MWM, EPM, and rotarod tests. All data were checked for normality using the Kolmogorov-Smirnov test. Analysis of variance (ANOVA) followed by Tukey’s post-hoc tests was performed. The one-way ANOVA model had the following assumptions: independence, normality, and homogeneity. Confidence level was 95%. Results are presented as mean ± standard error of the mean. P-values of 0.05 or lower were considered statistically significant.

## Results

MWM test results

**Figure 1:**
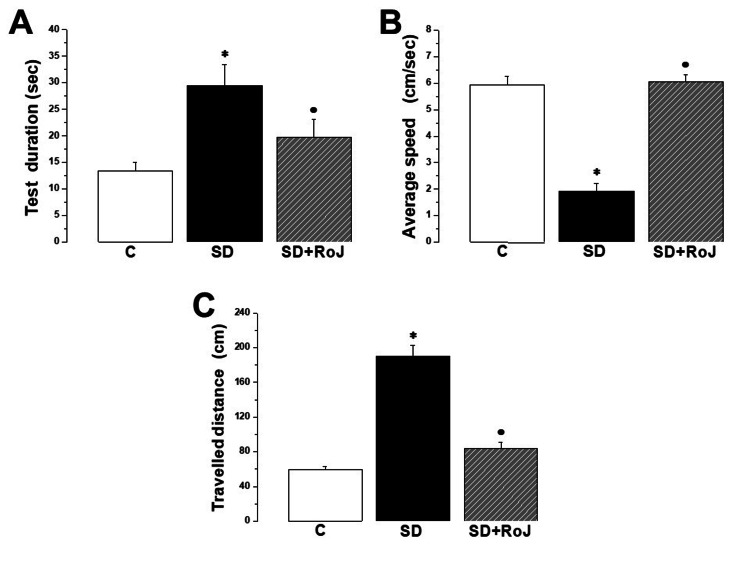
MWM test results demonstrate enhancement of spatial memory and learning in the SD+RoJ group (A) Test duration, (B) average speed, (C) traveled distance in control (C), sleep deprivation (SD), and SD treated with Rose of Jericho (SD+RoJ) groups. Number of rats in each group was 10. P-value ≤0.05 was considered significant. * indicates significance when compared to the C group. · indicates significance when compared to the SD group.

The MWM results demonstrated that SD significantly increased test duration (29.51 ± 3.57 sec) and decreased average speed (1.91 ± 0.3 sec) compared to corresponding values in the C group (13.41 ± 1.57 sec and 5.93 ± 0.34 sec, respectively). The SD+RoJ group showed improved test performance, as evidenced by reducing test duration (19.75 ± 3.36 sec) and increasing speed (6.06 ± 0.27 sec) when compared to the corresponding values in the SD group (p < 0.05). Additionally, the SD+RoJ group showed a significant reduction in total distance traveled (83.68 ± 6.83 cm) when compared to the SD group (190.38 ± 12.21 cm) (Figure 1).

EPM test results

**Figure 2:**
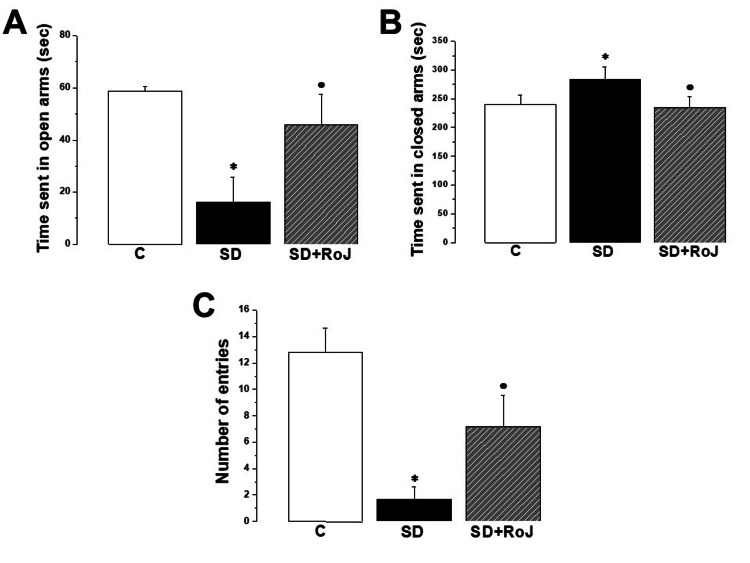
EPM test results reveal anxiolytic effect of the SD+RoJ group (A) Time spent in open arms, (B) time spent in closed arms, (C) number of control entries (C), sleep deprivation (SD), and SD treated with Rose of Jericho (SD+RoJ) groups. Number of rats in each group was 10. P-value ≤0.05 was considered significant. * indicates significance when compared to the C group. · indicates significance when compared to the SD group.

As shown in Figure 2, the EPM test results revealed significant behavioral differences across the study groups. Rats in the C group spent an average of 59.82 ± 3.29 sec in the open arms, while their counterparts in the SD group spent significantly less time (16.21 ± 9.44 sec, p ≤ 0.05). The SD+RoJ group spent significantly longer in the open arms when compared to the rats in the SD group (45.83 ± 11.64 sec, p ≤ 0.05). Understandably, C rats spent less time in the closed arms when compared to the SD rats (240.23 ± 16.73 vs. 283.81 ± 22.14 sec, respectively, p ≤ 0.05). The SD+RoJ group significantly decreased the time spent in the closed arms compared to the SD group (234.22 ± 19.62 sec, p ≤ 0.05). The number of entries dropped from 12.83 ± 1.82 in the C group to 1.61 ± 0.91 in the SD group (p ≤ 0.05), while it increased significantly in the SD+RoJ group (7.15 ± 2.39 entries) when compared to the SD group (p ≤ 0.05).

Rotarod test results

**Figure 3:**
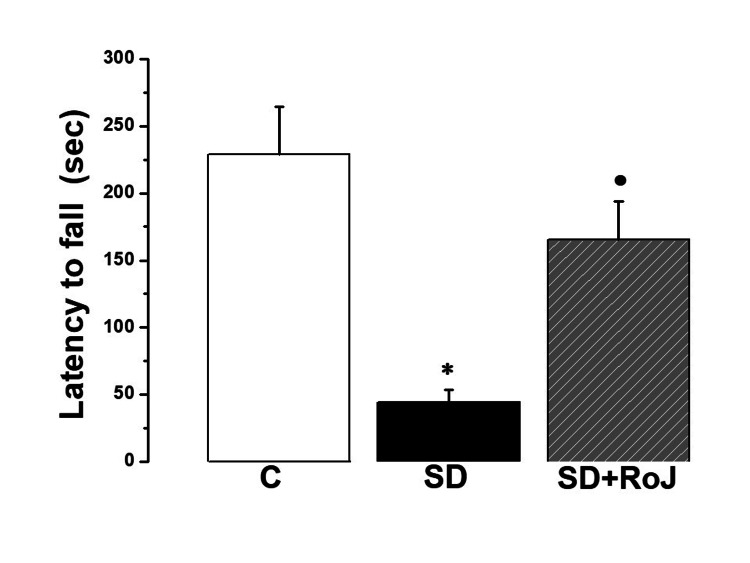
Rotarod test results show improvement of motor coordination in the SD+RoJ group Latency to fall in control (C), sleep deprivation (SD), and SD treated with Rose of Jericho (SD+RoJ) groups. Number of rats in each group was 10. P-value ≤0.05 was considered significant. * indicates significance when compared to the C group. · indicates significance when compared to the SD group.

The rotarod test results showed significant differences in motor coordination among the study groups. The latency in the C group was 228.67 ± 35.44 sec, significantly longer than the corresponding value in the SD group (44.2 ± 9.51 sec, p ≤ 0.05). Rats in the SD+RoJ showed significant improvement, as evidenced by a significantly longer latency when compared to the SD group (165.18 ± 28.77 sec, p ≤ 0.05) (Figure 3).

## Discussion

Sleep is a fundamental and recurring biological process in living species, playing a vital role in physical restoration and mental recovery [18]. While interest in natural substances like RoJ for cognitive health and mood regulation is growing, research on its effects on SD is limited, hindering evidence-based interventions. In the present study, the SD+RoJ group demonstrated potential beneficial effects on memory, learning, and behavior; RoJ could possess memory-boosting and anxiolytic effects. To our knowledge, our study is the first to report such therapeutic capacities of RoJ; hence, it substantially contributes to addressing the knowledge gap in SD's non-pharmacological interventions.

Our findings revealed that SD rats exhibited significant impairments in spatial memory, as evidenced by increased test duration, reduced average speed, and increased traveled distance in the MWM test. These deficits were effectively ameliorated by RoJ treatment, suggesting a potential role of ROJ in improving cognitive function in individuals experiencing SD. Sleep has beneficial effects on long-term memory (LTM). LTM patterns necessitate optimal sleep duration between learning and retrieval. Specifically, memory researchers have focused on the role of sleep in memory consolidation [19]. Neural activity during slow-wave or rapid eye movement sleep contributes to the consolidation process of memory [20]. Consequently, SD could deleteriously impact declarative and non-declarative memory consolidation [21]. Previous studies reported that SD induces hippocampal dysfunction with resultant memory deficits. Furthermore, they postulated that oxidative stress and inflammation could be the underlying mechanisms [22,23]. Interestingly, RoJ has been shown to possess antioxidant and anti-inflammatory properties. Their properties were effective in wound healing and improving fertility parameters [10,11]. Although the effect of RoJ on SD-dependent memory deficits has not been well documented, natural herbs such as chamomile (*Matricaria chamomilla* L.) and St. John's wort (*Hypericum perforatum*) have been shown to improve sleep latency and overall quality, as well as to enhance cognitive parameters [24,25].

In the present study, SD rats displayed increased anxiety- and depression-like behaviors while performing the EPM test. Animals in the SD group spent less exploration time in the open arms, more exploration time in the closed arms, and a lesser number of entries when compared to the C group. Treatment with RoJ significantly improved rats' EPM test performance, suggesting a potential anxiolytic/antidepressant effect. SD is typically related to negative neurobehavioral consequences, modification of behavioral patterns, and modulation of psychiatric disorders. Sleep-induced anxiogenesis, as a concept, is well-established [26]. Furthermore, it has been suggested that reduced sleep quantity increases the risk of major depression. The latter itself increases the risk of SD [27]. The necessity to establish a biological framework to reveal the interrelationship between SD and anxiety is crucial to establishing potential therapeutic targets for reducing sleep-induced anxiogenesis. Specific brain neurotransmitter mechanisms, such as the adenosinergic receptor system, have been postulated [28]. Interestingly, the link between oxidative stress and anxiety disorders is more evident in disorders associated with underlying inflammation. Disruption of physiological pathways induces oxidative stress that contributes to neuroinflammation and, subsequently, anxiety [29]. Therefore, the supremacy of RoJ in the treatment of SD-induced anxiety could arise from its antioxidant and anti-inflammatory properties.

In this study, SD rats demonstrated lower motor coordination when compared to the C group and SD+RoJ group, significantly reducing the SD rats from such deleterious consequences. SD-induced deterioration in motor and neurocognitive performance involves decreased physical performance, increased mental fatigue, and changes in neurotransmitter release. The latter could limit the learning capacity and storage and retrieval of the learned material. This might be explained, in part, by alterations in use-dependent synaptic plasticity [30].

Oxidative stress triggers multiple mechanisms that could affect motor and cognitive integrity. These mechanisms include mitochondrial dysfunction, neuronal death, neuroinflammation, and neurodegeneration. Thus, mitigating oxidative stress with potent antioxidants could prevent or at least slow down the progression of the catastrophic consequences of SD [31].

Limitations

The results of the present study are mainly descriptive and lack mechanistic insights. However, they could serve as a logical proof of concept. Further research is needed to confirm these findings and to elucidate the possible underlying mechanisms and molecular targets. Additionally, further work should optimize the RoJ therapeutic dosage and conduct translational studies in humans.

## Conclusions

The present study provides compelling evidence that RoJ can effectively mitigate SD's cognitive and behavioral impairments. To our knowledge, our study is the first to demonstrate the potential therapeutic effects of the RoJ in treating SD-induced deterioration of memory and learning, anxiogenesis, and neurocognitive incoordination. RoJ could improve cognitive function in individuals experiencing SD or related conditions. The anxiolytic effects of RoJ observed in this study may have clinical implications for individuals suffering from sleep-related anxiety. Furthermore, given its positive effects on motor coordination, RoJ could be investigated as a complementary therapy for conditions affecting motor function, such as Parkinson's disease or stroke.

## References

[1] Zielinski MR, McKenna JT, McCarley RW (2016). Functions and mechanisms of sleep. AIMS Neurosci.

[2] Potter GD, Skene DJ, Arendt J, Cade JE, Grant PJ, Hardie LJ (2016). Circadian rhythm and sleep disruption: causes, metabolic consequences, and countermeasures. Endocr Rev.

[3] Chattu VK, Manzar MD, Kumary S, Burman D, Spence DW, Pandi-Perumal SR (2018). The global problem of insufficient sleep and its serious public health implications. Healthcare (Basel).

[4] Evbayekha EO, Aiwuyo HO, Dilibe A, Nriagu BN, Idowu AB, Eletta RY, Ohikhuai EE (2022). Sleep deprivation is associated with increased risk for hypertensive heart disease: a nationwide population-based cohort study. Cureus.

[5] Azad MC, Fraser K, Rumana N, Abdullah AF, Shahana N, Hanly PJ, Turin TC (2015). Sleep disturbances among medical students: a global perspective. J Clin Sleep Med.

[6] Shaik L, Cheema MS, Subramanian S, Kashyap R, Surani SR (2022). Sleep and safety among healthcare workers: the effect of obstructive sleep apnea and sleep deprivation on safety. Medicina (Kaunas).

[7] Saintila J, Soriano-Moreno AN, Ramos-Vera C, Oblitas-Guerrero SM, Calizaya-Milla YE (2023). Association between sleep duration and burnout in healthcare professionals: a cross-sectional survey. Front Public Health.

[8] Chaachouay N, Zidane L (2024). Plant-derived natural products: a source for drug discovery and development. Drugs and Drug Candidates.

[9] Almundarij TI, Alharbi YM, Abdel-Rahman HA, Barakat H (2021). Antioxidant activity, phenolic profile, and nephroprotective potential of anastatica hierochuntica ethanolic and aqueous extracts against CCl(4)-induced nephrotoxicity in rats. Nutrients.

[10] Zin SR, Kassim NM, Alshawsh MA, Hashim NE, Mohamed Z (2017). Biological activities of Anastatica hierochuntica L.: a systematic review. Biomed Pharmacother.

[11] Rizk NI, Rizk MS, Mohamed AS, Naguib YM (2020). Attenuation of sleep deprivation dependent deterioration in male fertility parameters by vitamin C. Reprod Biol Endocrinol.

[12] Abdelhakeem E, Hashem MM, Farag MA, Alsofany JM (2024). Unlocking the wound healing potential of Anastatica hierochuntica L. (Kaff Maryam) Extract's nanosuspension: UPLC-MS/MS metabolic profiling, formulation development and statistical optimization. J Drug Deliv Sci Technol.

[13] Council NR (2011). Guide for the Care and Use of Laboratory Animals: Eighth Edition.

[14] Alqhtani MM, Al Mousa NA, Al Zayer NM, Al Abbas LA, Alamer N, Almousa MA, Naguib YM (2024). Safflower improves memory, learning, and behavior in rats subjected to sleep deprivation. Cureus.

[15] Vorhees CV, Williams MT (2006). Morris water maze: procedures for assessing spatial and related forms of learning and memory. Nat Protoc.

[16] Walf AA, Frye CA (2007). The use of the elevated plus maze as an assay of anxiety-related behavior in rodents. Nat Protoc.

[17] Carter RJ, Morton J, Dunnett SB (2001). Motor coordination and balance in rodents. Curr Protoc Neurosci.

[18] Killgore WD (2010). Effects of sleep deprivation on cognition. Prog Brain Res.

[19] Paller KA, Creery JD, Schechtman E (2021). Memory and sleep: how sleep cognition can change the waking mind for the better. Annu Rev Psychol.

[20] Rasch B, Born J (2013). About sleep's role in memory. Physiol Rev.

[21] Kim T, Kim S, Kang J, Kwon M, Lee SH (2022). The common effects of sleep deprivation on human long-term memory and cognitive control processes. Front Neurosci.

[22] Piber D (2021). The role of sleep disturbance and inflammation for spatial memory. Brain Behav Immun Health.

[23] Silva RH, Abílio VC, Takatsu AL (2004). Role of hippocampal oxidative stress in memory deficits induced by sleep deprivation in mice. Neuropharmacology.

[24] Kazemi A, Shojaei-Zarghani S, Eskandarzadeh P, Hashempur MH (2024). Effects of chamomile (Matricaria chamomilla L.) on sleep: a systematic review and meta-analysis of clinical trials. Complement Ther Med.

[25] Valvassori SS, Borges C, Bavaresco DV (2018). Hypericum perforatum chronic treatment affects cognitive parameters and brain neurotrophic factor levels. Braz J Psychiatry.

[26] Pires GN, Bezerra AG, Tufik S, Andersen ML (2016). Effects of acute sleep deprivation on state anxiety levels: a systematic review and meta-analysis. Sleep Med.

[27] Roberts RE, Duong HT (2014). The prospective association between sleep deprivation and depression among adolescents. Sleep.

[28] Chellappa SL, Aeschbach D (2022). Sleep and anxiety: from mechanisms to interventions. Sleep Med Rev.

[29] Fedoce AD, Ferreira F, Bota RG, Bonet-Costa V, Sun PY, Davies KJ (2018). The role of oxidative stress in anxiety disorder: cause or consequence?. Free Radic Res.

[30] Christova M, Aftenberger H, Nardone R, Gallasch E (2018). Adult gross motor learning and sleep: is there a mutual benefit?. Neural Plast.

[31] Dash UC, Bhol NK, Swain SK (2024). Oxidative stress and inflammation in the pathogenesis of neurological disorders: Mechanisms and implications. Acta Pharmaceutica Sinica B.

